# Relationship between dyslipidemia and carotid plaques in a high‐stroke‐risk population in Shandong Province, China

**DOI:** 10.1002/brb3.473

**Published:** 2016-04-22

**Authors:** Te Mi, Shangwen Sun, Guoqing Zhang, Yaser Carora, Yifeng Du, Shougang Guo, Mingfeng Cao, Qiang Zhu, Yongxiang Wang, Qinjian Sun, Xiang Wang, Chuanqiang Qu

**Affiliations:** ^1^ Neurology DepartmentShandong Provincial Hospital Affiliated to Shandong UniversityJinanShandong250021China; ^2^ Cardio‐Cerebrovascular Control and Research CenterInstitute of Basic MedicineShandong Academy of Medical SciencesJinanShandong250062China; ^3^ Neurology DepartmentWeifang Chinese Medicine HospitalWeifangShandong261041China; ^4^ Neurosurgery DepartmentWayne State University School of MedicineDetroitMichigan48201China; ^5^ Medical DepartmentShandong Provincial Hospital Affiliated to Shandong UniversityJinanShandong250021China

**Keywords:** Carotid plaque, dyslipidemia, stroke

## Abstract

**Introduction:**

The precise associations between stroke and carotid plaques and dyslipidemia are unclear. This population‐based study aimed to examine the relationship between carotid plaques and dyslipidemia in a high‐stroke‐risk population.

**Methods:**

Ultrasonography of left and right carotid arteries was conducted in 22,222 participants in a second screening survey of individuals with high stroke risk. Subjects were divided into two groups according to the presence or absence of carotid plaques. Blood TC (total cholesterol), TG (total triglycerides), and LDL‐C (low‐density lipoprotein cholesterol) levels were recorded.

**Results:**

Multivariate logistic regression analysis, controlled for gender, age, education, geographic region, smoking, exercise, and overweight (Model 2), identified TG as a predictor of carotid‐plaque risk (odds ratio [OR] = 1.109, 95% confidence interval [CI]: 1.038–1.185, *P *=* *0.002), and the association between carotid plaques and LDL‐C (OR = 0.967, 95%CI: 0.949–0.994, *P *=* *0.019) was less significant, whereas there was no association between carotid plaques and TC (OR = 1.002, 95%CI: 0.932–1.007, *P *=* *0.958). After additional adjustment for hypertension, diabetes, and atrial fibrillation (Model 3), TG remained a risk factor for carotid plaques (OR = 1.086, 95%CI: 1.016–1.161, *P *=* *0.015), but no associations were observed between carotid plaques and LDL‐C (OR = 0.972, 95%CI: 0.910–1.038, *P *=* *0.394) or TC (OR = 1.003, 95%CI: 0.933–1.079, *P *=* *0.928). Only the association between TG and carotid plaques (OR = 1.084, 95%CI: 1.014–1.159, *P *=* *0.017) was independent of all covariates (covariates in Model 3 plus history of stroke or transient ischemic attack, and stroke family history) in Model 4.

**Conclusion:**

These findings indicate that TG was an independent risk factor for carotid plaques in high‐risk population for stroke, whereas LDL‐C and TC were not associated with the appearance of carotid plaques independently.

## Introduction

The World Health Organization Monitoring Trends and Determinants in Cardiovascular Disease (MONICA) project has shown that the incidence of stroke in China is rising at an annual rate of 8.7%. About 30% of cases of cardiovascular disease end in death, whereas most of the survivors have hemiplegia, aphasia, and other disabilities (Bermúdez 2011). In response to this issue, the Stroke Screening and Prevention Program of the National Health and Family Planning Commission of China was tasked with refining the guidelines for stroke screening and prevention, and informing the public and medical professionals about the modifiable risk factors for stroke. This public health crisis highlighted the need to determine the risk factors for cerebrovascular diseases.

Carotid plaques are frequently observed in patients who have suffered a stroke (Gomez 1990). Carotid plaques have been reported to predict the development of atherosclerotic disease in patients (Stein and Johnson 2010), and stroke risk is strongly correlated with the presence of carotid plaques, irrespective of their locations (Hollander et al. 2002). Elevated levels of atherogenic lipoproteins and dyslipidemia are associated with an increased plaque thrombotic potential (Pan et al. 1997; Zawadzki et al. 2007). Dyslipidemia includes elevated levels of serum TC (total cholesterol), TG (total triglycerides), and LDL‐C (low‐density lipoprotein cholesterol), and reduced levels of HDL‐C (high‐density lipoprotein cholesterol). Studies in Japan identified high‐serum TC as a risk factor for ischemic stroke, specifically large‐artery occlusive infarction (Cui et al. 2012) and low TC (<160 mg/dL) as a critical risk factor for hemorrhagic stroke (Suzuki et al. 2011). Ridker et al. found that non‐HDL‐C and the ratio of TC to HDL‐C were as good as or even better than apolipoprotein fractions for predicting future cardiovascular events (Ridker et al. 2005). A systematic review and meta‐analysis suggested that statins can lower LDL‐C concentrations by an average of 1.8 mmol/L, which reduces the risk of stroke by 17% (Law et al. 2003). These previous studies thus suggest the existence of some correlations between dyslipidemia and stroke. Dyslipidemia is correlated with carotid artery disease and is a cerebrovascular disease risk factor (Gardener et al. 2009; Chang et al. 2013). However, despite evidence linking dyslipidemia with carotid‐plaque formation, no trials have investigated the association in large‐scale population studies in China. As a result, guidelines for stroke screening and prevention are not necessarily specific to the Chinese population. This study therefore aimed to explore the association between dyslipidemia and carotid plaques in a high‐stroke‐risk population in China. Assessment of this relationship will help to elucidate the pathophysiological association between elevated lipoproteins and carotid intima atherogenesis, and also help to refine the stroke screening and prevention guidelines in China.

## Subjects and Methods

### Ethics statement

This study was conducted according to the guidelines of the Helsinki Declaration. Ethical approval was obtained from the Ethics Committee of Shandong Provincial Hospital affiliated to Shandong University before the start of the study. Written informed consent was obtained from all the participants.

### Subjects

**Figure 1:**
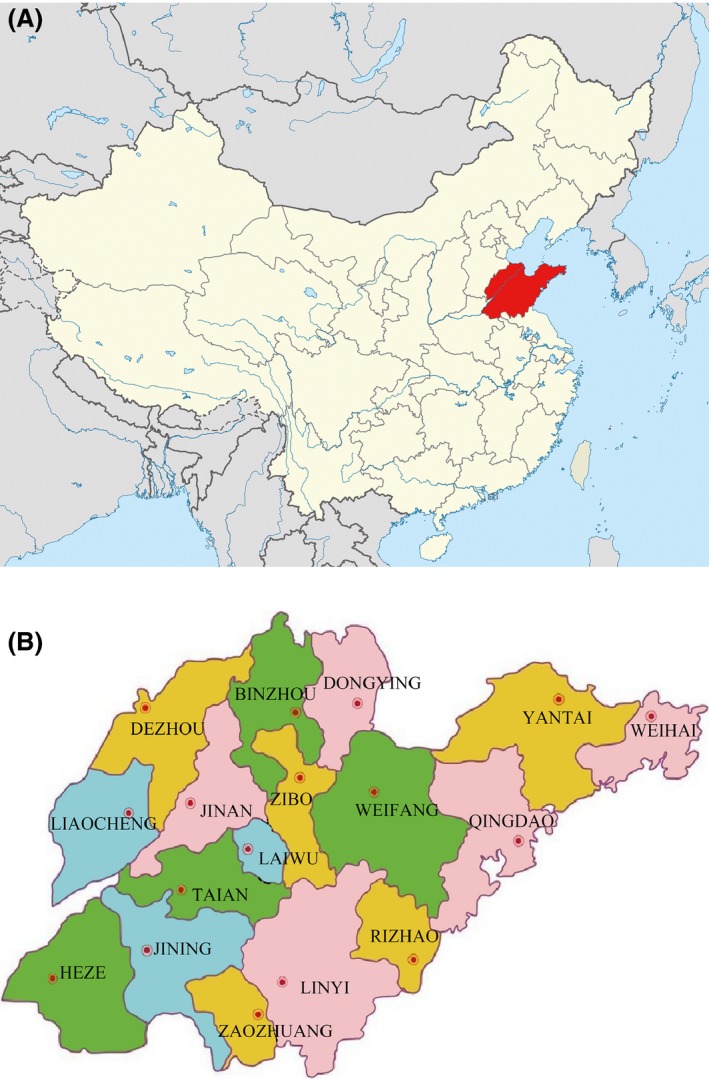
(A) Shandong Province in China. (B) The sampling spots in Shandong Province.

The Stroke Screening and Prevention Program of the National Health and Family Planning Commission of China implemented stroke screening in urban and rural residents in eastern China from 2011 to 2012. As a representative province of eastern China, Shandong is the second most populous province, with good economic development, comprising 17 districts (Fig. 1) and 139 county‐level administrative units. One urban and one rural location were selected randomly from each district, with a minimum of 6000 subjects per location. The initial pool of subjects included 231,289 permanent residents over the age of 40 years (birth date between January 1, 1937 and December 31, 1971). Residents who had lived in the location for >6 months were also included in the initial screen.

High‐risk‐stroke populations were selected based on a holistic assessment (the National Health Development Planning Commission Stroke Screening and Prevention Engineering) of their risk factors during every stage of the screening. Nine risk factors were described: hypertension, defined as a history of high blood pressure (≥140/90 mmHg) reported by the respondent or current use of antihypertensive medication; AF (atrial fibrillation), either reported by the respondent or defined as an irregular pulse during physical exam; diabetes mellitus, defined as a previous diagnosis, treatment with insulin or oral hypoglycemic medications, fasting plasma glucose ≥126 mg/dL, or glycosylated hemoglobin ≥6.5%; dyslipidemia, defined as current use of antilipidemic medication, TC ≥5.70 mmol/L, serum TGs ≥1.70 mmol/L, or LDL‐C ≥ 3.10 mmol/L; smoking, defined as current or former practice of smoking; and lack of exercise, defined as physical exercise <3 times a week for <30 min each (industrial and agricultural labor was considered exercise; overweight, defined as body mass index ≥26 kg/m^2^; and self‐reported family history of stroke). Subjects with at least three of these risk factors or a previous history of stroke or TIA (transient ischemic attack) were classified as a high‐risk population. High‐risk subjects who were not using any drugs that might interfere lipid concentrations were included in this study.

### Ultrasonography

Subjects at high risk of stroke underwent carotid artery ultrasound performed by physicians with at least 5 years of ultrasound experience. Subjects were laid in the supine position with their head turned to the side. Intima‐media thickness was observed for the presence of plaques, and the number of plaques in each vessel was counted. Carotid plaques were considered to be present if the intima‐media thickness was 1.2–1.4 mm (Chang et al. 2013).

### Statistical analysis

Questionnaire results and the results of physical and cognitive tests were recorded to calculate and compare the levels of risk factors in participants with and without high stroke risk. Normality distributions of continuous variables were tested by Kolmogorov–Smirnov tests. Continuous variables were represented as mean ± standard deviation, or median ± interquartile range. Discrete variables were represented as number (proportion). Differences between groups were tested by *t*‐test or Wilcoxon's rank sum tests for continuous or graded variables, or by *χ* ^2^ tests for discrete variables. Multivariate logistic regressions were used to explore the relationships between high stroke risk and all the risk factors. All reported *P‐*values were two sided, and *P *<* *0.05 was considered statistically significant.

## Results

The descriptive characteristics of the high‐stroke‐risk population are listed in Table 1. Among the 22,222 participants, 10,897 (49.0%) were men, which corresponded to the gender ratio in the elderly population as a whole. Compared with men, women had higher levels of TG, TC, and LDL‐C. Higher proportions of women also had hypertension, diabetes, AF or valvular heart disease, lack of exercise, overweight, and history of TIA compared with men, whereas men were more likely than women to have a previous stroke history and to smoke. There were significant differences between the genders for all risk factors except family history of stroke.

**Table 1:** The descriptive characters of the participants

	Total	Male	Female	
	(*n* = 22,222)	(*n* = 10,897)	(*n* = 11,325)	*P* a
Age, years	63 (10)	62 (13)	64 (13)	5.4469E‐22
Total triglycerides	1.79 (1.50)	1.36 (1.05)	1.52 (1.11)	1.3074E‐39
Total cholesterol	4.96 (1.54)	4.78 (1.45)	5.16 (1.64)	1.329E‐120
Low‐density lipoprotein cholesterol	3.03 (1.01)	2.84 (1.21)	3.06 (1.32)	1.7654E‐62
Education				
Primary school or under	13,884 (62.5%)	5728 (52.6%)	8156 (72.0%)	1.783E‐199
Middle school	5127 (23.1%)	3100 (28.4%)	2027 (17.9%)	
High school	2230 (10%)	1384 (12.7%)	846 (7.5%)	
College and above	981 (4.4%)	685 (6.3%)	296 (2.6%)	
Region of urban	11,303 (50.9%)	5655 (51.9%)	5648 (49.9%)	0.003
History of stroke	3720 (16.7%)	2163 (19.8)	1557 (13.7%)	4.0443E‐34
History of transient ischemic attack	5044 (22.7%)	2228 (20.4%)	2816 (24.9%)	3.772E‐15
Hypertension	16,016 (72.1%)	7665 (70.2%)	8361 (73.8%)	2.7632E‐09
Atrial fibrillation or valvular heart disease	3412 (15.4%)	1474 (13.5%)	1938 (17.1%)	1.2391E‐13
Smoking	6324 (28.5%)	5763 (52.9%)	561 (5%)	–
Diabetes	4595 (20.7%)	1919 (17.6%)	2676 (23.6%)	1.66E‐28
Activity	8797 (39.6%)	3948 (36.2%)	4849 (42.8%)	1.0485E‐23
Overweight	10,950 (49.3%)	4823 (44.3%)	6127 (54.1%)	1.0061E‐48
Family history of stroke	6156 (27.7%)	3022 (27.7%)	3134 (27.7%)	1
Number of carotid plaques				
0	5189 (23.4%)	2230 (20.5%)	2959 (26.1%)	1.8845E‐44
1	7422 (33.4%)	3455 (31.7%)	3967 (35.0%)	
≥2	9611 (43.2%)	5212 (47.8%)	4399 (43.8%)	

Values are mean (SD) or percentages.

a Student's *t‐*test was used for comparison of mean values, and *x* ^*2*^ test was used for comparison of proportions.

Univariate analysis was used to compare the characteristics of participants with and without carotid plaques (Table 2). Male sex, older age, smoking, rural location, lower education level, hypertension, diabetes, AF or valvular heart disease, history of stroke, and TIA were associated with increased likelihood of carotid plaques. Compared with those without carotid plaques, participants with carotid plaques had slightly lower LDL‐C (3.08 ± 1.25 vs. 3.02 ± 1.06 for without or with carotid plaques, respectively, *P *=* *0.001). There were no significant differences in TG and TC in relation to the presence or absence of carotid plaques (*P *=* *0.17 and *P *=* *0.76, respectively).

**Table 2:** The comparison of variables between the participants with and without carotid plaques

	Carotid plaques		*P*
	Without (*n* = 5189)	With (*n* = 17,033)	
Age, years	60 (12)	64 (13)	9.5174E‐152
Total triglycerides, mmol/L	1.44 (1.05)	1.44 (1.08)	0.174722429
Total cholesterol, mmol/L	4.96 (1.64)	4.96 (1.55)	0.760657295
Low‐density lipoprotein cholesterol, mmol/L	3.00 (1.29)	2.95 (1.27)	0.001331253
Gender (male)	2230 (42.98)	8867 (50.96)	1.93258E‐23
Education levels			
Primary school or under	3065 (59.1%)	10,819 (63.5%)	1.7833E‐199
Middle school	1336 (25.7%)	3791 (22.3%)	
High school	553 (10.7%)	1677 (9.8%)	
College or University	227 (4.4%)	733 (4.3%)	
Graduate and above	8 (0.2%)	13 (0.1%)	
Region of urban	2938 (56.6%)	8365 (49.1%)	2.72027E‐21
History of Stroke	633 (12.2%)	3087 (18.1%)	1.39859E‐23
History of Stroke	633 (12.2%)	3087 (18.1%)	1.39859E‐23
History of transient ischemic attack	1088 (21.0%)	3956 (23.2%)	0.000674552
Hypertension	3437 (66.2%)	12,579 (73.9%)	9.77148E‐27
Atrial fibrillation or Valvular heart disease	752 (14.5%)	2660 (15.6%)	0.049
Diabetes	973 (18.8%)	3622 (21.3%)	9.08212E‐05
Smoking	1305 (25.1%)	5019 (29.5%)	1.60141E‐09
Activity	2082 (40.1%)	6715 (39.4%)	0.367
Overweight	2916 (56.2%)	8034 (47.2%)	4.73955E‐30
Family history of Stroke	1569 (30.2%)	4587 (26.9%)	3.15893E‐06

Multivariate logistic regression analysis was used to determine if dyslipidemia was associated with the presence of carotid plaques. As shown in Table 3, controlled for gender, age, education, geographic region, smoking, exercise, and overweight (Model 2) identified TG as a predictor of carotid‐plaque risk (odds ratio [OR] = 1.109, 95% confidence interval [CI]: 1.038–1.185, *P *=* *0.002), and the association between carotid plaques and LDL‐C (OR = 0.967, 95% CI: 0.949–0.994, *P *=* *0.019) was less significant, whereas there was no association between carotid plaques and TC (OR = 1.002, 95% CI: 0.932–1.007, *P *=* *0.958). After additional adjustment for hypertension, diabetes, and AF (Model 3), TG remained a risk factor for carotid plaques (OR = 1.086, 95% CI: 1.016–1.161, *P *=* *0.015), but no associations were observed between carotid plaques and LDL‐C (OR = 0.972, 95% CI: 0.910–1.038, *P *=* *0.394) or TC (OR = 1.003, 95% CI: 0.933–1.079, *P *=* *0.928). Only the association between TG and carotid plaques (OR = 1.084, 95% CI: 1.014–1.159, *P *=* *0.017) was independent of all covariates (covariates in Model 3 plus history of stroke or TIA, and stroke family history) in Model 4.

**Table 3:** The association of carotid plaques and dyslipidemia

	Total triglycerides		Total cholesterol		Low‐density lipoprotein cholesterol	
	OR	*P*	OR	*P*	OR	*P*
Crude model	0.995 (0.933–1.060)	0.858	0.968 (0.903–1.038)	0.365	0.930 (0.873–0.990)	0.024
Model 1	0.999 (0.978–1.020)	0.907	0.991 (0.922–1.065)	0.799	0.935 (0.877–0.998)	0.043
Model 2	1.109 (1.038–1.185)	0.002	1.002 (0.932–1.077)	0.958	0.967 (0.949–0.994)	0.019
Model 3	1.086 (1.016–1.161)	0.015	1.003 (0.933–1.079)	0.928	0.972 (0.910–1.038)	0.394
Model 4	1.084 (1.014–1.159)	0.017	1.010 (0.939–1.086)	0.786	0.976 (0.914–1.043)	0.475

Model 1: controlling for age and gender.

Model 2: controlling for age, gender, education, regions, smoking, activity, and overweight.

Model 3: controlling for age, gender, education, regions, smoking, activity, overweight, hypertension, diabetes, and atrial fibrillation.

Model 4: controlling for all covariates in Model 3 plus history of stroke or transient ischemic attack, and stroke family history.

## Discussion

Eastern China is a densely populated and economically developed area. The standardized prevalence of stroke in adults aged 35 years old and above was 1469 per 100,000, which was much higher than in Central (1085 per 100,000) and Western (615 per 100,000) regions of China in 2002 (Zhai et al. 2009). Screening of stroke risk factors was implemented in Eastern China to improve the management of the high‐stroke‐risk population. The current population‐based study demonstrated that TG was an independent risk factor for carotid plaques in high‐risk population for stroke, whereas LDL‐C and TC were not associated with the appearance of carotid plaques independently.

Carotid plaques were more common among men and older subjects, and among those with a past medical history of hypertension, AF, or diabetes, and a history of smoking, stroke, or TIA, consistent with the results of previous studies. Compared with younger individuals, older individuals were more likely to suffer from cerebrovascular events because of increased plaque vulnerability associated with lipid accumulation (Fabris et al. 1994; Gonçalves et al. 2003; Grufman et al. 2014), and carotid plaques have also previously been associated with increased age (Redgrave et al. 2010). Although men are usually considered to have a lower risk of carotid plaques in the general population (Kofoed et al. 2002; Timóteo et al. 2013), men in this study accounted for a higher proportion of subjects with carotid plaques; this apparent discrepancy may be explained by the fact that our investigation was carried out in a high‐stroke‐risk population, rather than in the general population. Furthermore, stroke‐risk factors such as hypertension (Kwon et al. 2009; Pierdomenico et al. 2013), AF (Calmarza et al. 2015), and diabetes (Cardoso et al. 2012) all predisposed individuals to carotid plaques. Smoking also increased the presence of carotid plaques, and has previously been correlated with an increased risk of atherosclerosis (Deng et al. 2011). Subjects in this study with carotid plaques were more likely to have a history of stroke and TIA, and such ischemic cerebrovascular events are frequently caused by thromboembolisms arising from carotid atherosclerotic plaques (Sacco et al. 1989).

Hyperlipidemia is a major risk factor for stroke, and dyslipidemia may be related to carotid plaques. Lipid control is an effective measure for preventing atherosclerosis and cardiovascular and cerebrovascular diseases. A previous study showed that lower LDL and higher HDL levels were associated with decreased risk of stroke (Lewis and Segal 2010). A Chinese study found that higher serum non‐HDL‐C concentrations were associated with an increased risk of stroke (Wu et al. 2013), whereas a Japanese study identified high‐serum TC as a risk factor for ischemic stroke (Cui et al. 2012), and TG was associated with atherosclerotic stroke only in patients with low levels of LDL‐C (Kim et al. 2012). However, this study is the first to explore the association between dyslipidemia and carotid plaques in a high‐stroke‐risk population in China, and showed that TG was independently associated with carotid plaques in individuals at high risk of stroke, and LDL‐C was slightly protective against carotid plaques, even after controlling for gender and age, whereas there was no independent association between carotid plaques and TC. The apparent discrepancies between this and previous studies may be explained by differences in the study populations (high‐stroke‐risk population or general population). Our finding of only a slight association between dyslipidemia and carotid‐plaque formation suggests that control of dyslipidemia in individuals with other stroke‐risk factors may have limited value in the prevention and control of stroke.

This study had several limitations. First, measurements of HDL were lacking and analysis of different dyslipidemia phenotypes was therefore not comprehensive. Second, only the presence, and not the size and nature, of the carotid plaques was considered. Last, the use of drugs other than lipid‐lowering agents was not analyzed.

In brief, the results of this study showed that plasma TG levels contributed to the development of carotid plaques, whereas TC and LDL‐C levels have no impact on carotid plaques in individuals with other stroke‐risk factors. These results suggest that control of dyslipidemia (TG, TC, and LDL‐C) might be of limited use in controlling carotid plaques and preventing stroke. Further investigations are needed to explore the relationship between dyslipidemia and the pathogenesis of carotid plaques in other ethnic groups.

## Conflict of Interest

There is no conflict of interest.
