# Relationship between dyslipidemia and carotid plaques in a high‐stroke‐risk population in Shandong Province, China

**DOI:** 10.1002/brb3.610

**Published:** 2016-12-19

**Authors:** Te Mi, Shangwen Sun, Guoqing Zhang, Yaser Carcora, Yifeng Du, Shougang Guo, Mingfeng Cao, Qiang Zhu, Yongxiang Wang, Qinjian Sun, Xiang Wang, Chuanqiang Qu

In Mi et al. (2016), the last name of the fourth author was spelled incorrectly as Yaser Carora. The author's correct name should be Yaser Carcora.
